# Attack-Related Anticipatory Anxiety Symptoms in Familial Mediterranean Fever: An Exploratory Cross-Sectional Study

**DOI:** 10.3390/healthcare14121635

**Published:** 2026-06-10

**Authors:** Altuğ Güner

**Affiliations:** Department of Rheumatology, Bursa City Hospital, 16110 Bursa, Türkiye; guner_88_8@hotmail.com

**Keywords:** familial Mediterranean fever, anxiety, quality of life, inflammation, biomarkers, cross-sectional studies

## Abstract

**Background and Objectives:** Familial Mediterranean fever (FMF) is a chronic autoinflammatory disease characterized by recurrent inflammatory attacks and a persistent psychosocial burden. Although generalized anxiety symptoms have been investigated in FMF, disease-specific anticipatory concerns related to recurrent attacks remain insufficiently understood. This study aimed to investigate the associations of attack-related anticipatory anxiety symptoms with clinical characteristics, quality of life, and composite inflammatory indices in FMF. **Materials and Methods:** This exploratory cross-sectional study included 38 adult patients with FMF. Attack-related anticipatory anxiety symptoms were assessed using an exploratory six-item questionnaire. Generalized anxiety and quality of life were evaluated using the Generalized Anxiety Disorder-7 (GAD-7) and Short-Form–12 (SF-12), respectively. Composite inflammatory indices including the C-reactive protein–albumin–lymphocyte (CALLY) index, log-CALLY, hemoglobin–albumin–lymphocyte–platelet (HALP) score, and systemic immune-inflammation index (SII) were calculated from routine laboratory parameters. **Results:** Attack-related anticipatory anxiety scores demonstrated a significant positive correlation with GAD-7 scores (r = 0.581, *p* < 0.001) and an inverse correlation with SF-12 mental component scores (r = −0.380, *p* = 0.019). Direct correlations between attack-related anticipatory anxiety scores and composite inflammatory indices were weak and not statistically significant. In subgroup analysis, a higher annual attack burden was associated with higher GAD-7 scores, higher CRP and serum amyloid A values, and lower CALLY, log-CALLY, and HALP values. Differences in attack-related anticipatory anxiety, SF-12 MCS, and SII between attack burden groups did not reach statistical significance. In multivariable linear regression analysis, GAD-7 score remained independently associated with attack-related anticipatory anxiety symptoms (β = 0.438, *p* = 0.010). **Conclusions:** Attack-related anticipatory anxiety symptoms may represent an exploratory psychosocial dimension of FMF associated mainly with generalized anxiety symptoms and impaired mental well-being. Composite inflammatory indices appeared more closely related to annual attack burden than to attack-related anticipatory anxiety. These findings should be interpreted cautiously and considered hypothesis-generating.

## 1. Introduction

FMF is the most common monogenic autoinflammatory disease and is characterized by recurrent episodes of fever and serosal inflammation [1,2]. The disease is primarily associated with mutations in the MEFV gene and is particularly prevalent in populations originating from the Mediterranean basin [1]. Although FMF attacks are generally self-limited, recurrent inflammatory episodes and persistent subclinical inflammation may lead to a substantial physical, social, and psychological burden over time [2,3]. In addition to attack-related morbidity, the unpredictable nature of FMF attacks may negatively affect daily functioning, social participation, occupational performance, and overall well-being.

Psychological manifestations in FMF have increasingly attracted attention in recent years. Previous studies have demonstrated impaired health-related quality of life, mood disturbances, anxiety, and depressive symptoms in patients with FMF compared with healthy individuals [4]. However, most available studies have primarily focused on generalized psychological symptoms rather than disease-specific emotional responses associated with recurrent and unpredictable inflammatory attacks. In routine clinical practice, many patients report persistent concerns regarding the unpredictability of attacks, possible attack triggers, social disruption, work impairment, and fear of developing future attacks even during attack-free periods. These recurrent anticipatory concerns may represent a distinct psychosocial dimension that may not be adequately captured by conventional generalized anxiety assessments.

The concept of attack-related anticipatory anxiety may therefore provide additional insight into the psychosocial burden of FMF. Similar psychosocial constructs, including illness uncertainty, fear of recurrence, hypervigilance, and fear-avoidance behaviors, have previously been described in chronic relapsing diseases and chronic symptom-related conditions [5,6,7]. Mishel’s illness uncertainty theory proposes that unpredictability and insufficient control over disease-related events may substantially contribute to persistent psychological distress in chronic illnesses [5]. Likewise, fear-of-recurrence models suggest that recurrent disease episodes may lead to ongoing anticipatory concerns, heightened symptom monitoring, behavioral avoidance, and persistent emotional distress even during clinically stable periods [6]. Vlaeyen and Linton further emphasized that anticipatory fear and hypervigilance may contribute to avoidance behaviors and the long-term psychosocial burden in chronic recurrent conditions [7]. Given the relapsing and unpredictable nature of FMF, recurrent attacks may similarly contribute not only to physical disability but also to chronic anticipatory stress, heightened bodily vigilance, and persistent emotional burden.

Increasing evidence further suggests that inflammatory and neuroimmune pathways may contribute to anxiety-related and depressive symptoms through bidirectional interactions between immune activation and neuropsychological processes [8,9]. Inflammatory cytokines and chronic immune activation may influence neurotransmitter systems, stress regulation pathways, and sickness behavior responses, potentially contributing to impaired mental well-being in chronic inflammatory diseases [8,9]. Such mechanisms may adversely influence mental quality of life and may potentially contribute to persistent psychological distress in FMF. However, the relationship between attack-related anticipatory concerns and inflammatory burden in FMF remains insufficiently understood.

Recently, composite inflammatory indices derived from routine laboratory parameters have emerged as practical markers reflecting systemic inflammatory and immunometabolic burden in chronic inflammatory diseases. Among these, the CALLY index integrates inflammatory, nutritional, and immune-related components into a single parameter [10]. Recent evidence has suggested that the CALLY index may reflect inflammatory burden across different clinical phases of FMF [11]. In ankylosing spondylitis, the CALLY index has also been reported to reflect systemic inflammatory burden rather than patient-reported disease activity [12]. Similarly, the systemic immune-inflammation index (SII) has been investigated as a marker of inflammatory burden and disease severity in both oncological and rheumatic conditions [13,14]. Nevertheless, the potential associations of composite inflammatory indices with psychosocial burden and attack-related anticipatory symptoms in FMF remain largely unexplored.

Therefore, the present exploratory study aimed to investigate the associations of attack-related anticipatory anxiety symptoms with clinical characteristics, generalized anxiety symptoms, quality of life, and composite inflammatory indices in patients with FMF. We additionally explored whether composite inflammatory indices such as CALLY, log-CALLY, and SII may reflect recurrent attack-related inflammatory and psychosocial burden in FMF.

## 2. Materials and Methods

This cross-sectional observational study was conducted in accordance with the Strengthening the Reporting of Observational Studies in Epidemiology (STROBE) statement. Adult patients diagnosed with FMF according to the Tel Hashomer criteria were consecutively recruited from the Rheumatology outpatient clinic of Bursa City Hospital [1,2,15]. Patients younger than 18 years of age and those with active infection, malignancy, severe psychiatric disorders, pregnancy, or concomitant inflammatory rheumatic diseases were excluded. Severe psychiatric disorders were excluded because such conditions could independently dominate anxiety-related outcomes and substantially confound the interpretation of attack-related anticipatory anxiety. This exclusion did not refer to mild or previously treated anxiety/depressive symptoms unless they were severe enough to impair reliable questionnaire completion or clinical assessment. During the study period, 50 patients with a previous diagnosis of FMF were screened for eligibility. Of these, 12 were excluded because they did not meet the inclusion criteria or met at least one exclusion criterion, and 38 eligible patients were invited to participate. A total of 38 patients provided written informed consent and were included in the final analysis.

Demographic and clinical characteristics including age, sex, body mass index (BMI), disease duration, annual attack number, colchicine use, biologic treatment status, comorbidities, and MEFV mutation characteristics were recorded. Comorbidities were extracted from medical records and included documented chronic medical conditions and previously diagnosed anxiety or depressive disorders when present. Annual attack number was defined as the number of clinically compatible FMF attacks reported by the patient during the preceding 12 months. Attack frequency was based on patient self-report at the study visit; a prospective attack diary was not used. For exploratory subgroup analyses, patients were categorized as having a low attack burden (0–2 attacks/year) or high attack burden (≥3 attacks/year). This cut-off was selected pragmatically to distinguish patients with recurrent attacks occurring at least every few months from those with absent or infrequent attacks, and it was not intended to represent a validated disease activity threshold. Active disease was defined as the presence of at least one clinically compatible FMF attack during the preceding 12 months. This variable was used only for descriptive characterization of the cohort and was not intended to represent a validated disease activity index. Laboratory parameters including C-reactive protein (CRP), albumin, complete blood count, and routine biochemical measurements were obtained from hospital records. MEFV variant status was obtained from hospital genetic testing records. Genetic analyses had been performed as part of routine clinical care using a standard MEFV variant panel, and the recorded variants were extracted from the patients’ medical files. Variants were reported descriptively and were not independently reclassified for pathogenicity in the present study.

The study protocol was approved by the Bursa City Hospital Clinical Research Ethics Committee (Approval No: 2025-KAEK-47; Meeting No: 2026-6; Decision No: 8). Written informed consent was obtained from all participants prior to enrollment, and the study was conducted in accordance with the ethical principles of the Declaration of Helsinki.

### 2.1. Assessment of Attack-Related Anticipatory Anxiety

Because no validated FMF-specific instrument assessing attack-related anticipatory anxiety currently exists, an exploratory investigator-developed six-item questionnaire was used to preliminarily assess recurrent attack-related anticipatory concerns commonly reported by FMF patients during routine clinical follow-up.

The exploratory questionnaire included items evaluating anxiety related to imminent attacks, uncertainty regarding attack timing, disruption of daily activities and social plans because of attack-related concerns, anxiety during travel or planning activities outside the home, fear triggered by possible prodromal symptoms, and avoidance behaviors associated with anticipated attacks.

Each item was scored using a 5-point Likert scale ranging from 0 to 4 (0 = never, 1 = rarely, 2 = sometimes, 3 = frequently, and 4 = almost always). Total scores ranged between 0 and 24, with higher scores indicating greater attack-related anticipatory anxiety symptom burden.

The questionnaire was used for exploratory research purposes only and was not intended as a formal psychometrically validated diagnostic or disease activity assessment tool.

### 2.2. Assessment of Anxiety and Quality of Life

Generalized anxiety symptoms were assessed using the GAD-7 questionnaire [16]. The Turkish validity and reliability of the GAD-7 have previously been established [17]. Quality of life was evaluated using the SF-12, including both physical component summary (PCS) and mental component summary (MCS) scores [18]. The Turkish version of the SF-12 has also demonstrated acceptable validity and reliability [19].

### 2.3. Composite Inflammatory Indices

The CALLY index was calculated using the following formula [10,11,12]:

CALLY = (albumin × lymphocyte count)/CRP

Because of skewed distribution, logarithmic transformation of the CALLY index (log-CALLY) was additionally analyzed.

The HALP index score was calculated as follows:

HALP = (hemoglobin × albumin × lymphocyte count)/platelet count

For HALP calculation, hemoglobin was expressed as g/dL, albumin as g/L, lymphocyte count as ×10^9^/L, and platelet count as ×10^9^/L.

The systemic immune-inflammation index (SII) was calculated using the following formula [13,14]:

SII = (platelet count × neutrophil count)/lymphocyte count

All indices were calculated using routine laboratory parameters obtained on the day of clinical assessment.

### 2.4. Study Outcomes

The primary outcome of the study was the exploratory attack-related anticipatory anxiety score. Secondary outcomes included generalized anxiety symptoms assessed using the GAD-7, quality of life assessed using the SF-12 PCS and MCS scores, and annual attack burden. Composite inflammatory indices, including CALLY, log-CALLY, HALP, and SII, as well as mutation-related subgroup comparisons, were considered exploratory outcomes.

### 2.5. Statistical Analysis

Statistical analyses were performed using IBM SPSS Statistics for Windows, version 26.0 (IBM Corp., Armonk, NY, USA). The study was designed and reported in accordance with STROBE recommendations for cross-sectional observational studies. Because this was an exploratory single-center study, the sample size was primarily determined by the number of eligible patients who could be recruited during the study period. A formal a priori sample size calculation was not performed. As a post hoc power consideration, the available sample size of 38 patients provides limited statistical power for detecting small-to-moderate associations and subgroup differences, particularly in mutation-based analyses. Therefore, correlation analyses, subgroup comparisons, and regression findings were interpreted as exploratory and hypothesis-generating rather than as confirmatory.

The distribution of continuous variables was assessed using the Shapiro–Wilk test together with histogram and Q–Q plot evaluations. Continuous variables were expressed as mean ± standard deviation and median (minimum–maximum) values because several variables demonstrated non-normal distribution. Categorical variables were presented as number and percentage.

Correlations between clinical variables, psychological measures, and composite inflammatory indices were evaluated using Spearman’s correlation coefficient. Comparisons between independent groups were performed using the independent-samples t-test or Mann–Whitney U test, as appropriate according to distribution characteristics. Categorical variables were compared using the chi-square test or Fisher’s exact test when necessary.

Exploratory subgroup analyses were additionally performed according to annual attack burden, mutation burden, and MEFV variant status. Because of skewed distribution, logarithmic transformation of the CALLY index (log-CALLY) was additionally analyzed.

Variables were selected for the multivariable linear regression model based on clinical relevance, conceptual relationship with attack-related anticipatory anxiety, and potential associations observed in univariate analyses. Given the relatively limited sample size, the number of predictors included in the final regression model was intentionally restricted to reduce overfitting risk and improve model stability. Multicollinearity among predictors in the regression model was assessed using tolerance and variance inflation factor (VIF) values. Model diagnostics were additionally examined using standardized residuals, Cook’s distance, and the Shapiro–Wilk test of residual normality.

Given the exploratory nature of the investigator-developed questionnaire and several subgroup and secondary correlation analyses, no formal correction for multiple comparisons was applied. Accordingly, *p*-values from secondary correlation analyses, subgroup comparisons, inflammatory index analyses, and mutation-related comparisons were interpreted cautiously and regarded as hypothesis-generating rather than as confirmatory. A two-tailed *p* value < 0.05 was considered statistically significant.

## 3. Results

The demographic and clinical characteristics of the study population are summarized in Table 1. A total of 38 patients with FMF were included in the study. The mean age was 37.97 ± 11.25 years, and 57.9% of the cohort were female. The mean BMI was 26.41 ± 4.87 kg/m^2^, and the median disease duration was 3.50 years (range, 1–30 years). The mean annual attack number was 4.95 ± 5.68, with a median of 2.00 attacks per year. According to this descriptive definition, active disease was present in 81.6% of patients. Colchicine use was observed in 68.4% of the cohort, whereas biologic therapy was being administered in 18.4% of patients. Amyloidosis and comorbid diseases were identified in 7.9% and 34.2% of patients, respectively (Table 1). Documented comorbidities included hypertension, diabetes mellitus, chronic kidney disease, obesity, coronary artery disease, and other chronic medical conditions.

The distribution of MEFV variants is presented in Table 2. Single variants were identified in 57.9% of patients, whereas multiple variants were present in 42.1% of the cohort. The most frequently recorded MEFV variants were M694V heterozygous (28.9%) and R202Q (21.1%), followed by V726A (15.8%), M694V homozygous (13.2%), and E148Q (10.5%). R202Q was interpreted descriptively because of its debated pathogenic significance. Other less frequent variants were identified in 10.5% of patients (Table 2).

Laboratory findings and composite inflammatory indices are summarized in Table 3. Median CRP and serum amyloid A levels were 3.55 mg/L and 0.94 mg/L, respectively. The median CALLY index, log-CALLY, HALP score, and SII values were 34.31, 1.53, 5.44, and 485.89, respectively.

Patient-reported outcomes and quality-of-life measures are presented in Table 4. The mean attack-related anticipatory anxiety score was 11.68 ± 6.93, with a median value of 12.00 (range, 0–24). Mean GAD-7 score was 10.47 ± 6.19. Mean SF-12 physical component score (PCS) and mental component score (MCS) were 38.71 ± 9.78 and 40.73 ± 9.18, respectively, indicating impaired physical and mental quality of life within the study cohort (Table 4).

Correlations between attack-related anticipatory anxiety scores and clinical/inflammatory variables are presented in Table 5. Attack-related anticipatory anxiety scores correlated positively with GAD-7 scores (r = 0.581, *p* < 0.001) and inversely with SF-12 MCS (r = −0.380, *p* = 0.019). Correlations with annual attack number, CRP, serum amyloid A, CALLY, log-CALLY, HALP, SII, and SF-12 PCS were weak and did not reach statistical significance.

Correlations of GAD-7 scores with selected clinical and inflammatory variables are summarized in Table 6. GAD-7 scores correlated positively with attack-related anticipatory anxiety scores and inversely with both SF-12 PCS and SF-12 MCS. Correlations with annual attack number, log-CALLY, and SII did not reach statistical significance.

Correlations between SF-12 physical and mental component scores and selected clinical/inflammatory variables are presented in Table 7. SF-12 mental component scores correlated inversely with annual attack number, attack-related anticipatory anxiety scores and GAD-7 scores. Similarly, SF-12 physical component scores correlated inversely with GAD-7 scores whereas the inverse correlation with annual attack number showed borderline statistical significance. Correlations of SF-12 scores with log-CALLY and SII were weak and not statistically significant.

Correlations among composite inflammatory indices are summarized in Table 8. CALLY and log-CALLY showed a strong positive correlation. Both CALLY-based indices correlated positively with HALP and inversely with SII, whereas the inverse correlation between HALP and SII did not reach statistical significance.

Comparisons according to annual attack burden are presented in Table 9. Patients with a high attack burden (≥3 attacks/year, n = 17) had higher median GAD-7 scores than those with a low attack burden (0–2 attacks/year, n = 21) (*p* = 0.029). Attack-related anticipatory anxiety scores were numerically higher in the high attack burden group, but this difference did not reach statistical significance (*p* = 0.109). SF-12 PCS showed a borderline difference between groups (*p* = 0.054), whereas SF-12 MCS did not differ significantly (*p* = 0.814). Regarding inflammatory parameters, patients with a high attack burden had higher CRP and serum amyloid A values (*p* = 0.013 and *p* = 0.001, respectively). CALLY, log-CALLY, and HALP values were lower in the high attack burden group (*p* = 0.013, *p* = 0.013, and *p* = 0.012, respectively). SII values were numerically higher in the high attack burden group but did not differ significantly (*p* = 0.187).

Additional mutation related subgroup summaries are provided in Appendix A for descriptive purposes only. In particular, the M694V homozygous subgroup included only five patients; therefore, these findings were not interpreted inferentially and should be regarded as anecdotal rather than conclusive.

Multivariable linear regression analysis for attack-related anticipatory anxiety score is presented in Table 10. Among the variables included in the final regression model, GAD-7 score remained the only independent predictor of attack-related anticipatory anxiety score (β = 0.438, 95% CI: 0.121 to 0.755, *p* = 0.010). In contrast, SF-12 mental component score and log-CALLY did not retain independent statistical significance in the multivariable model. The final regression model demonstrated an adjusted R^2^ value of 0.268 (Table 10). Multicollinearity diagnostics did not indicate problematic collinearity among the predictors included in the final model. Tolerance values ranged from 0.764 to 0.931, and VIF values ranged from 1.074 to 1.309. Standardized residuals ranged from −2.15 to 1.84, and all Cook’s distance values were below 1.0, with a maximum value of 0.152. The residuals did not show a marked deviation from normality based on the Shapiro–Wilk test (*p* = 0.310).

Additional exploratory correlations between composite inflammatory indices and clinical and psychological variables are summarized in Appendix A (Table A1). Overall, lower CALLY and log-CALLY values together with higher SII levels tended to be associated with greater inflammatory and attack-related disease burden. However, direct associations between composite inflammatory indices and psychological measures remained limited.

**Figure 1:**
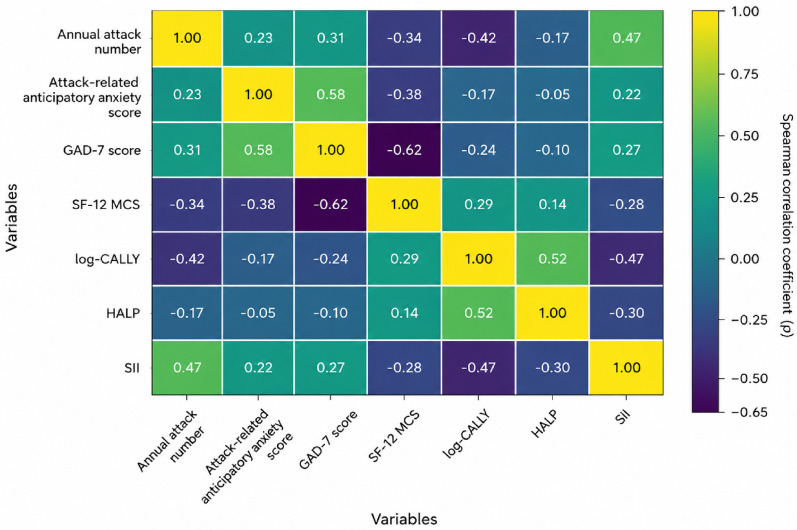
Correlation heatmap of clinical, psychological, and inflammatory variables in patients with familial Mediterranean fever.

Attack-related anticipatory anxiety demonstrated positive correlations with generalized anxiety symptoms and inverse correlations with mental quality-of-life scores. Annual attack number showed positive correlations with systemic immune-inflammation index (SII) and inverse correlations with log-CALLY values and mental quality of life. Overall, recurrent attack burden appeared to be associated with both increased inflammatory activity and greater psychosocial impairment in patients with FMF (Figure 1).

**Figure 2:**
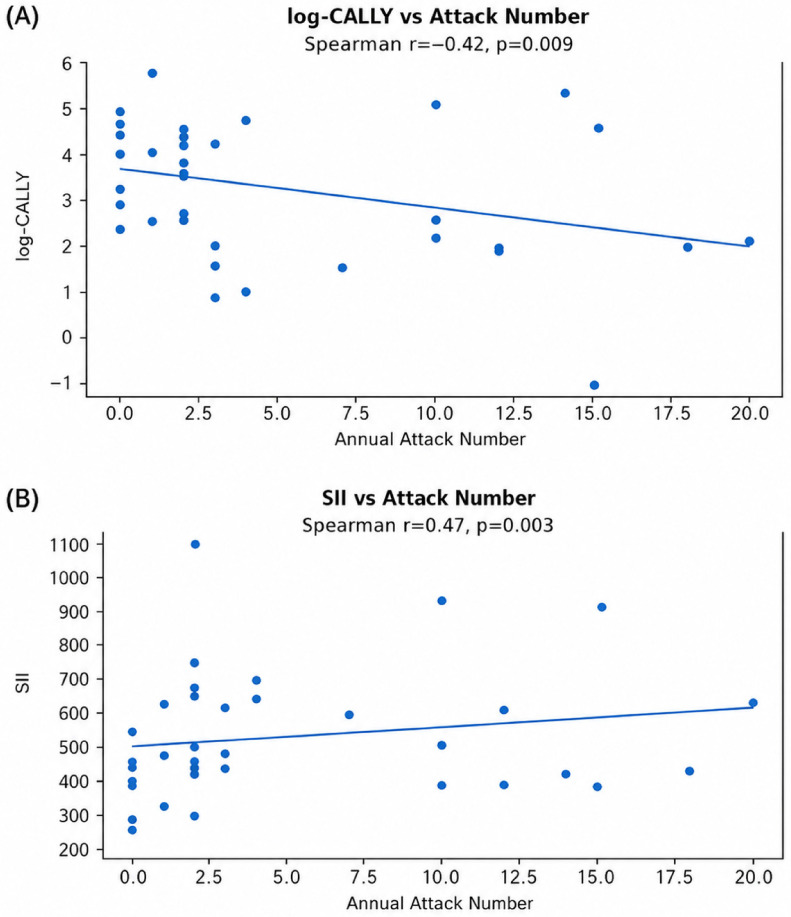
(**A**,**B**) Scatter plots demonstrating the relationships between annual attack number and composite inflammatory indices in patients with familial Mediterranean fever. (**A**) Annual attack number demonstrated an inverse association with log-CALLY. (**B**) Annual attack number showed a positive relationship with SII. Each point represents an individual participant, and solid lines indicate the overall regression trend.

**Figure 3:**
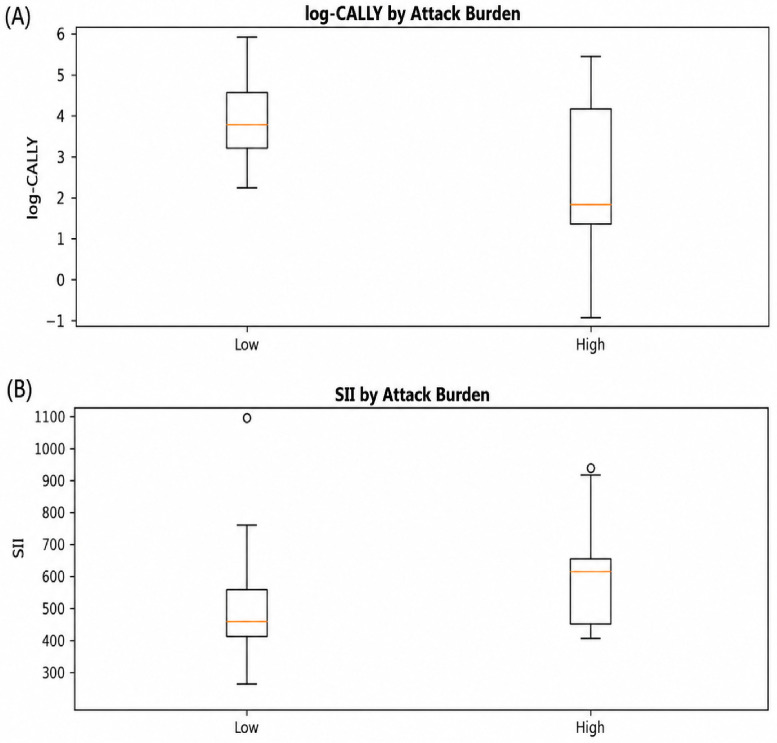
(**A**,**B**) Boxplots showing the distribution of composite inflammatory indices according to annual attack burden groups in patients with familial Mediterranean fever. (**A**) Distribution of log-CALLY values in low versus high annual attack burden groups. (**B**) Distribution of systemic immune-inflammation index (SII) values in low versus high annual attack burden groups.

Patients with a higher annual attack burden demonstrated lower log-CALLY values and higher SII levels compared with patients with a lower attack burden (Figure 2A,B). Boxes represent interquartile ranges, horizontal lines indicate median values, and whiskers indicate minimum and maximum values excluding outliers. These exploratory findings suggest a possible association between recurrent attack burden and inflammatory activity in FMF, but they should be confirmed in larger longitudinal studies (Figure 3A,B).

## 4. Discussion

The present cross-sectional STROBE-compliant study investigated the relationships between attack-related anticipatory anxiety symptoms, generalized anxiety symptoms, quality of life, and composite inflammatory indices in patients with FMF. The principal findings of the present study were as follows: (i) attack-related anticipatory anxiety symptoms demonstrated a strong positive association with generalized anxiety symptoms and an inverse association with mental quality of life; (ii) a higher annual attack burden was associated with worse psychological outcomes and increased inflammatory burden; (iii) lower CALLY and log-CALLY values together with higher SII levels were associated with a greater attack burden; and (iv) although inflammatory indices were associated with attack-related disease burden, their direct associations with psychological measures remained relatively limited.

FMF is characterized not only by recurrent inflammatory attacks but also by a substantial psychosocial burden related to disease unpredictability [1,2,3,4]. Previous studies have demonstrated impaired quality of life, anxiety, depressive symptoms, and mood disturbances in patients with FMF compared with healthy individuals [4]. However, most previous studies primarily evaluated generalized psychological symptoms rather than disease-specific anticipatory concerns. In routine clinical practice, many patients with FMF report persistent anxiety regarding the possibility of future attacks, uncertainty about attack timing, fear triggered by minor bodily symptoms, and concerns regarding disruption of work, travel, or social activities even during attack-free periods. In this context, the present study focused on attack-related anticipatory anxiety and found that these symptoms were associated with generalized anxiety and poorer mental quality of life.

Importantly, the investigator-developed questionnaire used in the present study was not intended as a formal psychometric instrument but rather as a preliminary symptom-oriented assessment of recurrent attack-related anticipatory concerns commonly encountered during routine clinical practice. Similar psychosocial constructs, including illness uncertainty, fear of recurrence, hypervigilance, and fear-avoidance behaviors, have previously been described in chronic relapsing diseases and chronic symptom-related conditions [5,6,7]. Nevertheless, because the questionnaire has not undergone formal psychometric validation, these findings should be considered preliminary and hypothesis-generating.

Patients with chronic rheumatic and autoinflammatory diseases are frequently exposed to persistent uncertainty regarding symptom recurrence, disease progression, and social functioning. In FMF specifically, the unpredictable timing and severity of attacks may lead patients to continuously monitor bodily sensations and modify daily activities because of a fear of future attacks. Such anticipatory concerns may gradually evolve into persistent hypervigilance and behavioral avoidance patterns, potentially contributing to long-term psychosocial burden even during attack-free periods. Therefore, attack-related anticipatory anxiety may represent a relevant psychosocial dimension in FMF, but this interpretation requires confirmation in larger longitudinal studies using validated FMF-specific tools.

The observed relationship between attack-related anticipatory anxiety symptoms and mental quality of life appears biologically and clinically plausible. Increasing evidence suggests that inflammatory and neuroimmune pathways may contribute to anxiety-related and depressive symptoms through bidirectional interactions between immune activation and neuropsychological processes [8,9]. Chronic inflammatory activation may influence neurotransmitter systems, stress regulation pathways, and sickness behavior responses, potentially contributing to impaired mental well-being in inflammatory diseases [8,9]. Although the present study did not identify strong direct correlations between inflammatory indices and psychological measures, the observed associations between attack burden, quality-of-life impairment, and inflammatory markers may still support the presence of a multidimensional interaction between disease activity and psychological well-being in FMF. However, these findings should not be interpreted as evidence of a direct biomarker–anxiety relationship.

Another important finding of the study was the relationship between recurrent attack burden and inflammatory parameters. In subgroup analysis, a higher annual attack burden was associated with higher CRP and serum amyloid A values and lower CALLY, log-CALLY, and HALP values. However, differences in attack-related anticipatory anxiety, SF-12 MCS, and SII did not reach statistical significance between attack burden groups. These findings suggest that recurrent attack burden may be accompanied by inflammatory and immunometabolic changes, although the subgroup results should be interpreted cautiously because of the exploratory design and limited sample size.

The CALLY index has recently emerged as a multidimensional marker integrating inflammatory, nutritional, and immune-related components [10]. Although initially investigated mainly in oncological settings, recent studies have increasingly explored its potential role in inflammatory rheumatic diseases [10,11,12]. Torun and Çigiloğlu recently reported that the CALLY index may reflect inflammatory burden across different clinical phases of FMF [11]. Similarly, our previous study in axial spondyloarthritis suggested that lower CALLY values may reflect systemic inflammatory burden rather than patient-reported disease activity [12]. The present findings appear generally consistent with these observations, as lower CALLY and log-CALLY values were associated with a greater annual attack burden and increased inflammatory activity in FMF.

In contrast to CALLY, the SII demonstrated positive associations with CRP, serum amyloid A, and annual attack frequency. SII has previously been associated with inflammatory burden and adverse outcomes in both oncological and rheumatic diseases [13,14]. SII showed a positive correlation with annual attack number in correlation analysis; however, SII did not differ significantly between low and high attack burden groups. This discrepancy may reflect the exploratory nature of the subgroup analysis, the limited sample size, and the loss of information when a continuous variable such as annual attack number is dichotomized. Therefore, SII-related findings should be interpreted cautiously. Interestingly, SII demonstrated inverse correlations with CALLY and log-CALLY, suggesting that these indices may capture different aspects of inflammatory and immunometabolic dysregulation. Nevertheless, the clinical relevance of these composite indices in FMF remains exploratory and requires validation in larger prospective cohorts.

Despite the associations between composite inflammatory indices and attack burden, direct relationships between inflammatory indices and psychological measures remained relatively limited. Although positive but statistically non-significant associations between SII, log-CALLY, and mental quality-of-life measures were observed, these findings did not reach statistical significance.

Psychological distress in FMF is likely multifactorial and may be influenced by disease unpredictability, previous attack experiences, social and occupational limitations, coping strategies, and individual psychological resilience. Therefore, the present findings should be viewed as preliminary evidence of a complex relationship among attack frequency, inflammatory burden, and psychosocial outcomes, rather than as evidence of a direct inflammatory marker–anxiety association.

The present study has several limitations that should be acknowledged. First, the cross-sectional design precludes causal inference regarding the relationships between inflammatory burden and psychological outcomes. Second, the relatively limited sample size and the number of exploratory analyses increase the risk of both type I and type II statistical errors. In particular, subgroup comparisons and secondary correlation analyses were underpowered and should not be interpreted as confirmatory findings. This issue was especially relevant for mutation-based subgroup analyses, particularly the M694V homozygous summary, because the subgroup sizes were very small and imbalanced. These findings were therefore retained only as descriptive observations and were not interpreted inferentially. Third, the investigator-developed six-item questionnaire used to assess attack-related anticipatory anxiety was intended as an exploratory symptom-oriented measure and has not undergone formal psychometric validation. Because item-level responses were not retained in the final analytic dataset, internal consistency analyses, including Cronbach’s α and corrected item–total correlations, could not be performed retrospectively. Similarly, factor structure, construct validity, test–retest reliability, responsiveness, and clinically meaningful cut-off values could not be evaluated in the present study. Therefore, all findings based on this questionnaire should be interpreted cautiously as exploratory and hypothesis-generating. In addition, annual attack frequency was assessed retrospectively and no prospective attack diary was used, which may have introduced recall bias. Therefore, findings related to this construct should be interpreted cautiously until validated FMF-specific assessment tools become available. In addition, no formal correction for multiple comparisons was applied because of the exploratory nature of several secondary and subgroup analyses. Accordingly, these findings should primarily be considered hypothesis-generating. Finally, inflammatory markers were evaluated at a single time point and may not fully reflect longitudinal inflammatory burden or temporal fluctuations in psychological symptoms. In addition, because patients with severe psychiatric disorders were excluded to reduce major confounding and ensure reliable questionnaire completion, the findings may not be generalizable to FMF patients with severe psychiatric comorbidity. Given the relatively large number of exploratory subgroup and secondary analyses relative to the sample size, the risk of false-positive findings should be carefully considered.

Despite these limitations, the study also has important strengths. To our knowledge, this is among the first studies specifically investigating attack-related anticipatory anxiety symptoms in FMF together with composite inflammatory indices and quality-of-life measures. In addition, the simultaneous evaluation of CALLY, log-CALLY, HALP, and SII may provide a broader perspective regarding inflammatory and immunometabolic burden in FMF. The study also addresses a clinically recognizable yet insufficiently investigated psychosocial dimension of FMF that may extend beyond conventional disease activity assessments.

## 5. Conclusions

In conclusion, in this exploratory cross-sectional study, attack-related anticipatory anxiety symptoms in FMF were associated with generalized anxiety symptoms and a poorer mental quality of life. A higher annual attack burden was also associated with lower CALLY/log-CALLY values and higher SII levels. However, direct associations between composite inflammatory indices and attack-related anticipatory anxiety were limited and did not reach statistical significance. Therefore, these findings should be interpreted as preliminary and hypothesis-generating rather than as evidence of causal relationships or clinical utility. Larger prospective studies using validated FMF-specific psychosocial assessment tools are needed to clarify the relationships among recurrent attack burden, inflammatory activity, quality of life, and attack-related anticipatory anxiety symptoms in FMF.

## Figures and Tables

**Table 1:** Demographic and Clinical Characteristics of the Study Population.

Variable	FMF Patients (n = 38)
Age (years)	37.97 ± 11.25; 39.50 (18–64)
Female sex, n (%)	22 (57.9)
Male sex, n (%)	16 (42.1)
BMI (kg/m^2^)	26.41 ± 4.87; 25.80 (18.7–38.4)
Disease duration (years)	9.34 ± 10.05; 3.50 (1–30)
Annual attack number	4.95 ± 5.68; 2.00 (0–20)
Active disease, n (%)	31 (81.6)
Colchicine use, n (%)	26 (68.4)
Biologic therapy, n (%)	7 (18.4)
Amyloidosis, n (%)	3 (7.9)
Comorbidity, n (%)	13 (34.2)

**Table 2:** MEFV Variant Characteristics of the FMF Cohort.

Variable	n (%)
Single variant	22 (57.9)
Multiple variants	16 (42.1)
M694V heterozygous	11 (28.9)
M694V homozygous	5 (13.2)
R202Q	8 (21.1)
V726A	6 (15.8)
E148Q	4 (10.5)
Other variants	4 (10.5)

R202Q was reported descriptively because its pathogenic significance remains controversial and it is often regarded as a polymorphism or a variant of uncertain clinical significance rather than a clearly pathogenic FMF-causing variant. Percentages for individual MEFV variants are not mutually exclusive.

**Table 3:** Laboratory Parameters and Composite Inflammatory Indices.

Variable	FMF Patients (n = 38)
CRP (mg/L)	3.55 (1.20–10.70)
Serum amyloid A (mg/L)	0.94 (0.34–1.89)
Albumin (g/L)	45.50 (44.00–47.58)
Lymphocyte count (×10^9^/L)	2.05 (1.74–2.44)
Hemoglobin (g/dL)	13.20 (11.45–14.82)
Platelet count (×10^9^/L)	249.00 (212.50–273.00)
Neutrophil count (×10^9^/L)	4.38 (3.60–5.20)
CALLY index	34.31 (7.74–82.28)
log-CALLY	1.53 (0.89–1.91)
HALP score	5.44 (3.84–6.66)
SII	485.89 (439.89–641.95)

Values are presented as median (interquartile range). CRP, C-reactive protein; CALLY, C-reactive protein–albumin–lymphocyte index; HALP, hemoglobin–albumin–lymphocyte–platelet score; SII, systemic immune-inflammation index.

**Table 4:** Patient-Reported Outcomes and Quality of Life Measures.

Variable	FMF Patients (n = 38)
Attack-related anticipatory anxiety score	11.68 ± 6.93; 12.00 (0–24)
GAD-7 score	10.47 ± 6.19; 8.50 (0–21)
SF-12 PCS	38.71 ± 9.78; 40.22 (21.27–53.13)
SF-12 MCS	40.73 ± 9.18; 41.10 (14.84–63.60)

**Table 5:** Correlations of Attack-related Anticipatory Anxiety Score with Clinical and Inflammatory Variables.

Variable	r	*p*
Annual attack number	0.232	0.160
Disease duration	0.030	0.857
CRP	0.192	0.249
Serum amyloid A	0.215	0.195
CALLY index	−0.109	0.516
log-CALLY	−0.174	0.297
HALP score	−0.054	0.748
SII	0.221	0.181
GAD-7	0.581	<0.001
SF-12 PCS	−0.224	0.177
SF-12 MCS	−0.380	0.019

**Table 6:** Correlations of GAD-7 Score with Selected Clinical and Inflammatory Variables.

Variable	r	*p*
Annual attack number	0.314	0.055
log-CALLY	−0.245	0.139
SII	0.268	0.104
Attack-related anticipatory anxiety	0.581	<0.001
SF-12 PCS	−0.371	0.022
SF-12 MCS	−0.622	<0.001

**Table 7:** Correlations of SF-12 Physical and Mental Component Scores with Selected Clinical and Inflammatory Variables.

Variable	PCS r (*p*)	MCS r (*p*)
Annual attack number	−0.319 (0.051)	−0.342 (0.036)
log-CALLY	0.244 (0.140)	0.287 (0.081)
SII	−0.261 (0.113)	−0.276 (0.093)
Attack-related anticipatory anxiety	−0.224 (0.177)	−0.380 (0.019)
GAD-7	−0.371 (0.022)	−0.622 (<0.001)

**Table 8:** Correlations Among Composite Inflammatory Indices.

Variable Pair	r	*p*
CALLY vs. log-CALLY	0.912	<0.001
CALLY vs. HALP	0.488	0.002
CALLY vs. SII	−0.441	0.006
log-CALLY vs. HALP	0.523	0.001
log-CALLY vs. SII	−0.474	0.003
HALP vs. SII	−0.298	0.069

**Table 9:** Comparison According to Annual Attack Burden.

Variable	Low Attack Burden (0–2/year), n = 21	High Attack Burden (≥3/year), n = 17	*p*
Attack-related anticipatory anxiety score	10.00 (4.00–15.00)	14.00 (10.00–19.00)	0.109
GAD-7 score	8.00 (5.00–13.00)	16.00 (8.00–18.00)	0.029
SF-12 PCS	44.12 (36.09–48.66)	34.05 (30.63–40.80)	0.054
SF-12 MCS	41.09 (39.08–45.15)	41.11 (36.76–43.70)	0.814
CRP (mg/L)	1.90 (1.00–4.10)	10.80 (1.60–17.90)	0.013
Serum amyloid A (mg/L)	0.46 (0.32–0.98)	1.89 (1.40–12.50)	0.001
CALLY index	46.97 (25.06–85.06)	7.02 (4.58–70.21)	0.013
log-CALLY	1.67 (1.40–1.93)	0.85 (0.66–1.85)	0.013
HALP score	5.84 (4.24–7.27)	4.19 (2.90–5.46)	0.012
SII	462.81 (420.36–561.86)	610.93 (451.87–645.64)	0.187

Values are presented as median (interquartile range). GAD-7, Generalized Anxiety Disorder-7; SF-12 PCS, Short-Form–12 Physical Component Summary; SF-12 MCS, Short-Form–12 Mental Component Summary; CRP, C-reactive protein; CALLY, C-reactive protein–albumin–lymphocyte index; HALP, hemoglobin–albumin–lymphocyte–platelet score; SII, systemic immune-inflammation index.

**Table 10:** Multivariable Linear Regression Analysis for Attack-related Anticipatory Anxiety Score.

Predictor	Β	95% CI	*p*	VIF
GAD-7	0.438	0.121 to 0.755	0.010	1.309
SF-12 MCS	−0.183	−0.512 to 0.146	0.260	1.244
log-CALLY	−0.126	−0.451 to 0.199	0.421	1.074

Adjusted R^2^ = 0.268.

## Data Availability

The datasets generated and/or analyzed during the current study are available from the corresponding author upon reasonable request.

## Abbreviations

The following abbreviations are used in this manuscript:

FMF	Familial Mediterranean Fever
GAD-7	Generalized Anxiety Disorder-7
SF-12	12-Item Short-Form Health Survey
PCS	Physical Component Summary
MCS	Mental Component Summary
CRP	C-reactive Protein
CALLY	C-reactive Protein–Albumin–Lymphocyte Index
HALP	Hemoglobin–Albumin–Lymphocyte–Platelet Score
SII	Systemic Immune-Inflammation Index
BMI	Body Mass Index
MEFV	Mediterranean Fever Gene
STROBE	Strengthening the Reporting of Observational Studies in Epidemiology
CI	Confidence Interval

## Appendix A. Supplementary Subgroup and Secondary Correlation Analyses

*p*-Values were obtained using Spearman’s rank correlation analysis for continuous variables and the Mann–Whitney U test for subgroup comparisons, as appropriate. Because of the exploratory nature of these analyses and the relatively limited sample size, no formal correction for multiple comparisons was applied in the primary analyses. Therefore, the findings presented in this appendix should be interpreted cautiously and considered hypothesis-generating.

**Table A1:** Exploratory Correlations of Composite Inflammatory Indices with Clinical and Psychological Variables.

Variable	CALLY r (*p*)	log-CALLY r (*p*)	HALP r (*p*)	SII r (*p*)
CRP	−0.681 (<0.001)	−0.724 (<0.001)	−0.248 (0.133)	0.522 (0.001)
Serum amyloid A	−0.594 (<0.001)	−0.633 (<0.001)	−0.206 (0.214)	0.441 (0.006)
Annual attack number	−0.388 (0.016)	−0.421 (0.009)	−0.171 (0.305)	0.474 (0.003)
Disease duration	0.098 (0.559)	0.112 (0.503)	−0.044 (0.792)	0.051 (0.761)
Attack-related anticipatory anxiety	−0.109 (0.516)	−0.174 (0.297)	−0.054 (0.748)	0.221 (0.181)
GAD-7	−0.182 (0.275)	−0.245 (0.139)	−0.101 (0.546)	0.268 (0.104)
SF-12 PCS	0.202 (0.224)	0.244 (0.140)	0.118 (0.480)	−0.261 (0.113)
SF-12 MCS	0.236 (0.153)	0.287 (0.081)	0.142 (0.394)	−0.276 (0.093)

Spearman’s rank correlation analysis.

**Table A2:** Exploratory Comparison According to Mutation Burden.

Variable	Single Variant (n = 22)	Multiple Variants (n = 16)	*p*
Attack-related anticipatory anxiety score	10.64 ± 6.46	13.12 ± 7.51	0.287
GAD-7 score	11.14 ± 5.65	9.56 ± 6.94	0.614
SF-12 PCS	41.33 ± 9.30	35.10 ± 9.53	0.053
SF-12 MCS	39.74 ± 10.33	42.09 ± 7.43	0.668
Annual attack number	5.18 ± 5.46	4.62 ± 6.13	0.443
CRP (mg/L)	18.20 ± 43.14	5.01 ± 4.60	0.605
Serum amyloid A	5.57 ± 9.14	0.97 ± 0.91	0.174
log-CALLY	1.30 ± 0.73	1.53 ± 0.55	0.383
HALP score	4.68 ± 1.77	6.26 ± 2.46	0.069
SII	579.03 ± 205.75	500.19 ± 119.85	0.416

Mann–Whitney U test.

**Table A3:** Descriptive Summary According to M694V Homozygous Status.

Variable	M694V Homozygous (−), n = 33	M694V Homozygous (+), n = 5
Attack-related anticipatory anxiety score	11.21 ± 6.84	14.80 ± 7.51
GAD-7 score	10.02 ± 6.11	13.60 ± 6.87
SF-12 PCS	39.62 ± 9.53	32.41 ± 9.41
SF-12 MCS	41.74 ± 8.91	33.81 ± 8.74
Annual attack number	4.41 ± 5.33	8.60 ± 7.43
log-CALLY	1.48 ± 0.60	0.79 ± 0.41
SII	571.42 ± 180.21	372.91 ± 64.40

Because the M694V homozygous subgroup included only five patients, this table is presented only as a descriptive, anecdotal subgroup summary. No inferential interpretation should be made from these findings.

**Table A4:** Exploratory Comparison According to M694V Heterozygous Status.

Variable	M694V Heterozygous (−)	M694V Heterozygous (+)	*p*
Attack-related anticipatory anxiety score	10.61 ± 6.59	14.45 ± 7.21	0.152
GAD-7 score	9.52 ± 5.74	13.00 ± 6.78	0.136
SF-12 PCS	40.84 ± 9.25	33.92 ± 9.33	0.064
SF-12 MCS	43.55 ± 8.22	33.61 ± 7.92	0.006
Annual attack number	4.03 ± 4.84	7.45 ± 7.03	0.118
log-CALLY	1.52 ± 0.59	1.00 ± 0.55	0.063
SII	485.32 ± 129.40	703.17 ± 197.51	0.027

Mann–Whitney U test. These genotype-specific analyses were exploratory and were not included among the primary study outcomes because of the relatively limited subgroup sample sizes.
